# Comparison of methods for texture analysis of QUS parametric images in the characterization of breast lesions

**DOI:** 10.1371/journal.pone.0244965

**Published:** 2020-12-31

**Authors:** Laurentius O. Osapoetra, William Chan, William Tran, Michael C. Kolios, Gregory J. Czarnota

**Affiliations:** 1 Department of Radiation Oncology, Sunnybrook Health Sciences Centre, Toronto, Ontario, Canada; 2 Department of Radiation Oncology, University of Toronto, Toronto, Ontario, Canada; 3 Physical Sciences, Sunnybrook Research Institute, Toronto, Ontario, Canada; 4 Department of Medical Biophysics, University of Toronto, Toronto, Ontario, Canada; 5 University of Waterloo, Toronto, Ontario, Canada; 6 Evaluative Clinical Sciences, Sunnybrook Research Institute, Toronto, Ontario, Canada; 7 Department of Physics, Ryerson University, Toronto, Ontario, Canada; University of Montreal, CANADA

## Abstract

**Purpose:**

Accurate and timely diagnosis of breast carcinoma is very crucial because of its high incidence and high morbidity. Screening can improve overall prognosis by detecting the disease early. Biopsy remains as the gold standard for pathological confirmation of malignancy and tumour grading. The development of diagnostic imaging techniques as an alternative for the rapid and accurate characterization of breast masses is necessitated. Quantitative ultrasound (QUS) spectroscopy is a modality well suited for this purpose. This study was carried out to evaluate different texture analysis methods applied on QUS spectral parametric images for the characterization of breast lesions.

**Methods:**

Parametric images of mid-band-fit (MBF), spectral-slope (SS), spectral-intercept (SI), average scatterer diameter (ASD), and average acoustic concentration (AAC) were determined using QUS spectroscopy from 193 patients with breast lesions. Texture methods were used to quantify heterogeneities of the parametric images. Three statistical-based approaches for texture analysis that include Gray Level Co-occurrence Matrix (GLCM), Gray Level Run-length Matrix (GRLM), and Gray Level Size Zone Matrix (GLSZM) methods were evaluated. QUS and texture-parameters were determined from both tumour core and a 5-mm tumour margin and were used in comparison to histopathological analysis in order to classify breast lesions as either benign or malignant. We developed a diagnostic model using different classification algorithms including linear discriminant analysis (LDA), *k*-nearest neighbours (KNN), support vector machine with radial basis function kernel (SVM-RBF), and an artificial neural network (ANN). Model performance was evaluated using leave-one-out cross-validation (LOOCV) and hold-out validation.

**Results:**

Classifier performances ranged from 73% to 91% in terms of accuracy dependent on tumour margin inclusion and classifier methodology. Utilizing information from tumour core alone, the ANN achieved the best classification performance of 93% sensitivity, 88% specificity, 91% accuracy, 0.95 AUC using QUS parameters and their GLSZM texture features.

**Conclusions:**

A QUS-based framework and texture analysis methods enabled classification of breast lesions with >90% accuracy. The results suggest that optimizing method for extracting discriminative textural features from QUS spectral parametric images can improve classification performance. Evaluation of the proposed technique on a larger cohort of patients with proper validation technique demonstrated the robustness and generalization of the approach.

## Introduction

Breast cancer demonstrates a high incidence and leads to high morbidity in women [1,2]. In 2017, there were 250,520 newly diagnosed cases of female breast cancer and 42,000 death in the United States [3]. Early detection of breast carcinoma through screening can enhance prognosis as appropriate treatments are provided to patients at an earlier stage of the disease [2]. For this purpose, accurate and precise diagnostic techniques are required.

Breast cancer diagnosis is based on clinical examination, imaging findings, and confirmed by histopathological results [2]. The current imaging workflow for breast cancer diagnosis begins with x-ray mammography, followed by standard ultrasound imaging (B-mode US imaging), dynamic contrast-enhanced magnetic resonance imaging (DCE-MRI) as needed, followed by core-needle biopsy, as required [2]. Mammography is susceptible to both providing false positive readings and concealing an underlying malignancy because of superimposition of normal breast parenchyma [4]. Biopsy remains as the gold standard for pathological confirmation of malignancy and tumour grade characterization [2]. However, as biopsies are invasive in nature, they are associated with pain and a hypothetical increased risk of tumour cell migration [5]. Furthermore, the low specificity of B-mode US images resulted in a trend of increasingly performing unnecessary biopsies [4,6]. The specificity of breast cancer detection may be increased using Dynamic Contrast-Enhanced Magnetic Resonance Imaging (DCE-MRI) [7]. However, DCE-MRI is not always available for rapid diagnosis purposes. The development of imaging techniques that can perform rapid and accurate characterization of breast lesions is highly beneficial for early detection of breast carcinoma and triaging patients in a screening workflow [8,9].

Previously, sonographic characteristics of solid breast nodules have been used in the characterization of breast lesions [10]. In addition, morphologic features of the breast tumours have been utilized for developing computer aided diagnosis (CAD) systems for the characterization of breast lesions using artificial neural network (ANN) [11,12]. Recently, deep learning approaches have also been applied for breast mass classification in sonography [13,14]. As these studies used B-mode US images, which are instrument- and operator-dependent, the sonographic features and other quantitative features derived from them are influenced by acquisition system settings. QUS spectroscopy may address these limitations. QUS spectroscopy estimate spectral-based parameters through analysis of raw radiofrequency (RF) signal and utilize normalization procedure to remove instrument-dependent effects [15,16]. Furthermore, attenuations because of propagation through intervening tissue layers and the tumour are also compensated prior to estimation of tumour scattering parameters. This results in the attenuation-corrected normalized power spectrum (NPS) or the backscatter coefficient (BSC) [16]. Linear parametrization of the attenuation-corrected NPS results in QUS spectral parameters including mid-band-fit (MBF), spectral-slope (SS), and 0-MHz intercept (SI) [17,18]. These parameters are linked to the scattering power, size, and shape of acoustic scatterers [19,20]. Furthermore, fitting of theoretical acoustic scattering models to the measured BSC allows for estimation of scatterer property estimates: average scatterer diameter (ASD) and average acoustic concentration (AAC) [20–22].

The utilities of QUS spectroscopy have been demonstrated in the assessment of tumour responses to cancer therapies both pre-clinically and clinically [23–27], characterization of different types of tissues such as prostate, liver, and retina [28–33], determination of blood-clot and various intravascular plaque components [34–36], and detection of tumour deposits in *ex vivo* lymph nodes [37]. Spontaneously occurring mammary fibroadenomas (benign lesions) and mammary carcinomas (malignant lesions) have also been differentiated using QUS techniques pre-clinically [21]. In addition, the methods have also been utilized to differentiate different types of mammary cancers including carcinoma and sarcoma [22]. Furthermore, QUS spectroscopy have also been used in clinical research to differentiate breast tumours from the surrounding normal tissues in patients with locally-advanced breast cancer (LABC) [38]. Recently, QUS spectral parametric imaging, along with texture and novel derivative texture analysis, have been used in the characterization of breast lesions [8,9].

Tumour micro-environment, physiology and metabolism exhibit spatial heterogeneities that offer diagnostic and prognostic values [39–43]. These have been demonstrated using different imaging modalities, such as MRI [44], positron emission tomography (PET) [45,46], computerized tomography (CT) [47,48], and diffuse optical spectroscopy (DOS) [26]. Texture analysis methods can quantify such heterogeneities [49]. Texture analysis using GLCM techniques has been applied to B-mode US images for breast lesions characterization, as benign and malignant lesions often demonstrate homogeneous and heterogeneous textures, respectively [50–54]. However, as these images are system- and operator-dependent, the quantitative texture measures do not represent independent intrinsic properties of a tumour. Application of texture analysis on QUS spectral parametric images alleviates this limitation, providing texture parameters that represent intrinsic tumour characteristics.

In earlier studies, we only used the GLCM method to analyze texture of QUS spectral parametric images [8,9]. In this study, different texture methods were applied to QUS spectral parametric images encompassing the breast mass and its 5-mm margin. As there are diverse approaches for analyzing texture, here we evaluated three statistical-based texture analysis methods that have been commonly used in the literature [55]. These include the Gray Level Co-occurrence Matrix (GLCM) [49], the Gray Level Run Length Matrix (GRLM) [56–60], and the Gray Level Size Zone Matrix (GLSZM) [61] methods. GLCM methodology quantifies texture using second-order statistics of gray scale image histograms [49,53–55]. The GRLM method characterizes texture images based on the run-length of image gray levels [56–60], whereas the GLSZM method measures the size of homogeneous zones for each gray level in an image [61]. These approaches were applied here on a larger cohort of 193 patients with breast lesions. QUS-based texture analysis of tumour margins has been demonstrated in the *a priori* prediction of response and survival in LABC patients undergoing neoadjuvant chemotherapy (NAC) [62]. Margin information is further potentially useful for characterizing breast lesions as has been shown recently using QUS spectroscopy [9], US Nakagami shape parameters and texture features of B-mode US images [63].

The study here developed a diagnostic model to classify breast lesions as either benign or malignant. Specifically, our work used the different texture methods described above along with standard and advanced classification algorithms that include linear discriminant analysis (LDA), *k*-nearest neighbours (KNN), support vector machine with radial basis function kernel (SVM-RBF), and a shallow artificial neural network (ANN). The performance of the diagnostic model using standard classification algorithms was evaluated using leave-one-out cross-validation (LOOCV) and split-sample/hold-out validation. Evaluation of the proposed approach on the independent hold-out testing set demonstrates the generalization of our model. On the other hand, ANN implementation partitioned the data into training, validation, and testing subsets and evaluated performance of the trained network on the hold-out testing set. Classification performance was assessed using the receiver operating characteristics (ROC) analysis to obtain metrics of sensitivity, specificity accuracy, AUC, positive predictive value (PPV), and negative predictive value (NPV). The ground truth about the nature of lesions as either benign or malignant was obtained from clinical patient reports comprising of MR images and biopsy results, for the biopsied lesions. The results suggest that QUS spectral parametric imaging, along with optimized texture analysis methods, is a potential imaging modality for the rapid, accurate, and non-invasive characterization of breast lesions.

## Methods

### Study protocol & data acquisition

This study was conducted based on institutional-research-ethics board approval (Sunnybrook Health Sciences Center). US RF Data were acquired from 193 patients (benign and malignant) with breast lesions at the Rapid Diagnostic Unit (RDU) of the Louise Temerty Breast Cancer Center at Sunnybrook Health Sciences Center, Toronto, Ontario, Canada upon obtaining written informed consent. Data acquisition was performed by an experienced sonographer using a Sonix Touch US system (Ultrasonix, Vancouver, Canada). The system was equipped with a linear array transducer (L14-5/60W) that operates at 6.5 MHz center frequency and 3–8 MHz bandwidth. Beam-formed RF data were digitized using 40 MHz sampling frequency. Data acquisition was performed along 512 scan lines, spanning a 6cm lateral field-of-view (FOV) and a 4cm depth, obtained using a high line density option. This feature allows acquisition of beamformed A-lines from 512 transmit-receive apertures through application of electronic time delay. The focal depth was set at the center of the tumour. US images were acquired at approximately 5-mm intervals across the tumour volume via hand translation.

A radiologist with experience in interpreting breast US images performed contouring of the tumour regions of interest (ROI) on B-mode US images. QUS spectroscopy and texture analyses were performed on selected ROIs covering tumour core and a 5-mm tumour margin. The margin is an extension of the tumour from the core up to a 5-mm maximum distance into the surrounding area (peri-tumoural region). A 5-mm margin was chosen as it previously provided the best characterization results in breast cancer patients in other QUS applications [62].

The inclusion criterion of this study is sonographically identified breast lesions after the masses has been identified on clinical examination, in combination with imaging findings. The ground truth lesions identification as either benign or malignant was obtained from clinical reports that include results from MR images and biopsy, for lesions that underwent biopsy. Patients were excluded if the lesion was not able to be identified during the US scan. The goal of this study is to demonstrate that QUS spectroscopy and different texture methods can extract imaging biomarkers that are distinct between benign and malignant breast lesions.

### Feature extraction: Linear regression & acoustic form-factor parameters

QUS spectral parametric images were created using a sliding window technique with a 2-mm by 2-mm kernel and a 94% window overlap between adjacent kernels in the axial and lateral directions. The kernel size was chosen to include enough number of acoustic wavelengths for reliable spectral estimation, while preserving image texture. At 6.5MHz center frequency, the kernel includes 8 wavelengths axially and 17 scan-lines laterally.

Individual RF scan lines within the window were gated along the beam direction using a Hanning function for spectral analysis. We used the Fast Fourier Transform (FFT) algorithm to extract the power spectrum of the sample. Several independent adjacent RF signals within the window were used to obtain an averaged power spectrum that better represents the true power spectrum of the sample. Normalization procedure was performed using the reference phantom technique to remove instrument-dependent effects and to account for transmission path factors [15,16,20]. The reference phantom was composed of 5–30 μm glass beads embedded in a homogeneous medium of oil droplets that were immersed in gelatin. The measured attenuation coefficient and speed of sound of the phantom were 0.786dB/cm/MHz and 1,540m/s, respectively (University of Wisconsin, Department of Medical Physics, Madison, WI, USA). Prior to estimating spectral parameters, attenuation correction was performed. We assumed an attenuation coefficient of 1dB/cm/MHz for the intervening breast tissues [64,65] and estimated the local attenuation coefficient of the tumour (ACE) using a spectral difference method [66]. It estimates the rate of change in the log-transformed spectral power magnitude over depth in the ROI (over the tumour region) relative to the reference phantom for each frequency within the analysis bandwidth [66]. The power of the frequency dependence of the attenuations was assumed to be linear over the analysis bandwidth [66]. Our choice of 2-mm by 2-mm kernel size and typical ROI lengths greater than 35λ satisfy the requirement for optimal attenuation estimation, prescribed by Labyed *et al*. [66]. Specifically, Labyed *et al*. concluded that window sizes greater than 5λ and ROI sizes greater than 35λ resulted in the mean and STD errors of the ACEs that are less than 15% and 10%, respectively [66]. The measured BSC from the sample *σ*_*m*_(*f*) was calculated using [16,20]

$$\sigma_{m}(f)=\sigma_{r}(f)\frac{{\left|S_{m}(f)\right|}^{2}}{{\left|S_{r}(f)\right|}^{2}}e^{\left\{4(\alpha_{m}-\alpha_{r})(R+\frac{\Delta z}{2})\right\}}\propto NPS(f),$$ (1)

where *σ*_*r*_(*f*) is the BSC of the reference phantom, *S*_*m*_(*f*) and *S*_*r*_(*f*) are the RF spectra from the sample and the reference phantom, respectively. Parameters *α*_*m*_ and *α*_*r*_ are the attenuation functions from the sample and the reference phantom, respectively. Parameter *R* is the distance from the transducer face to the proximal side of the ROI window, and Δz is the kernel length. The MBF, SS, and SI parameters were obtained from linear regression analysis of the attenuation-corrected NPS. Subsequently, using more complex acoustic scattering models of soft tissues, acoustic scattering parameters can be obtained. We fitted theoretical BSC from spherical Gaussian acoustic form factor model *σ*_*theor*_(*f*) to the measured BSC to obtain average scatterer diameter (ASD) *a*_*eff*_ and average acoustic concentration (AAC) *n*_*z*_ parameters [20,67]. The AAC represented the net scattering strength [19,21,22,67]. It is defined as the product of average number density of scatterers $\overset{-}{n}$ and squared of the fractional difference in the acoustic impedance between the scatterer and surrounding tissue $\gamma_{0}^{2}$ [19,21,22,67]. The theoretical BSC is given as [20,67]

$$\sigma_{theor}(f)=Cf^{4}a_{eff}^{6}n_{z}F(f,a_{eff}),$$ (2)

where $C=\frac{\pi^{2}}{36c_{l}^{4}}$ and *c*_*l*_ is the speed of sound. *F*(*f*, *a*_*eff*_) is the form factor that captures the frequency dependence of the scattering. These analyses resulted in parametric images of MBF, SS, SI, ASD, and AAC. From these images, mean-value parameters were obtained from tumour core and tumour margin. Core and margin analyses were reflected in core-to-margin ratio (CMR) and core-to-margin-contrast ratio (CMCR) metrics in order to compare pixel intensities between the two regions:

$$CMR=\frac{mean({ROI}_{Core})}{std({ROI}_{Margin})}$$ (3)

$$CMCR=\frac{\left|mean({ROI}_{Core})-mean({ROI}_{Margin})\right|}{\frac{1}{2}(std({ROI}_{Core})+std({ROI}_{Margin}))}$$ (4)

[9]. Mean-value parameters, CMR, and CMCR parameters of each parametric image were subsequently used as potential features for classification.

### Feature extraction: Texture analysis methods

#### GLCM (Gray Level Co-occurrence Matrix) method

The GLCM method realizes second-order statistical analysis by studying the spatial relationship between neighbouring pixels in an image [49]. The full range of gray levels in each parametric image was linearly scaled into 16 discrete gray levels. We evaluated symmetric GLCM matrices from each parametric image at inter-pixel distances: 1, 2, 3, 4, 5 pixels and at four angular directions: 0°, 45°, 90°, and 135°. From these GLCM matrices, we extracted GLCM features that include:

$$Contrast=\sum_{i,j=0}^{N_{g}}{\left|i-j\right|}^{2}p(i,j)$$ (5)

$$Correlation=\frac{1}{\sigma_{i}\sigma_{j}}\sum_{i,j=0}^{N_{g}}\left(i-\mu_{i})(j-\mu_{j})p(i,j\right)$$ (6)

$$Energy=\sum_{i,j=0}^{N_{g}}p^{2}(i,j)$$ (7)

$$Homogeneity=\sum_{i,j=0}^{N_{g}}\frac{p(i,j)}{1+|i-j|}$$ (8)

In Eqs 5–8, *p*(*i*, *j*) is the gray level matrix element that represents the probability of having neighbouring pixels with intensities *i* and *j* in the image. *N*_*g*_ denotes the number of gray levels, while *μ* and *σ* are the mean and standard deviation for row *i* or column *j* of the GLCM matrix. Textural features are subsequently averaged over distances and angular directions. Textural measures were assumed to be reflected in these averaged values [49]. Contrast quantifies local gray level variations in the parametric image. Smoother image results in lower contrast, while coarser image produces higher contrast. Correlation represents linear correlation between neighbouring pixels. Energy measures textural uniformity between neighbouring pixels, while homogeneity quantifies the incidence of pixel pairs of different intensities [8].

#### GRLM (Gray Level Run-length Matrix) method

The GRLM method characterizes the coarseness of texture based on run-length of image gray levels [56–60]. A gray level run is a set of consecutive, collinear pixels having the same gray level *i* in a prescribed direction *θ* (flat zone) [56–60]. For a given image, the size of the run-length matrix is the number of gray levels *N*_*G*_ by the number of run-length *N*_*R*_. A run-length matrix element *p*_*RL*_(*i*, *j*|*θ*) is defined as the number of runs of *j* pixels with gray level intensities *i* in the direction *θ*. The GRLM method was applied on each parametric image. Each parametric image was quantized into 16 discrete gray levels prior to texture estimation. Subsequently, run-length matrices were evaluated for directions *θ* = 0°, 45°, 90°, and 135°. From each run-length matrix, we can extract run-length features [56–60]. In the following, let the total number of run-length in the image $s=\sum_{i=1}^{N_{G}}\sum_{j=1}^{N_{R}}p_{RL}(i,j|\theta )$, while $\mu_{i}=\sum_{i=1}^{N_{G}}\sum_{j=1}^{N_{R}}p_{RL}(i,j|\theta )i$, $\mu_{j}=\sum_{i=1}^{N_{G}}\sum_{j=1}^{N_{R}}p_{RL}(i,j|\theta )j$, and $r(j|\theta )=\sum_{i=1}^{N_{G}}p_{RL}(i,j|\theta )$.

Short Run Emphasis (SRE):

$$SRE=\frac{1}{s}\sum_{i=1}^{N_{G}}\sum_{j=1}^{N_{R}}\frac{p_{RL}(i,j|\theta )}{j^{2}}=\frac{1}{s}\sum_{j=1}^{N_{R}}\frac{r(j|\theta )}{j^{2}}$$ (9)

Long Run Emphasis (LRE):

$$LRE=\frac{1}{s}\sum_{i=1}^{N_{G}}\sum_{j=1}^{N_{R}}j^{2}p_{RL}(i,j|\theta )=\frac{1}{s}\sum_{j=1}^{N_{R}}j^{2}r(j|\theta )$$ (10)

Gray Level Nonuniformity (GLN):

$$GLN=\frac{1}{s}\sum_{i=1}^{N_{G}}{\left(\sum_{j=1}^{N_{R}}p_{RL}(i,j|\theta )\right)}^{2}$$ (11)

Run Length Nonuniformity (RLN):

$$RLN=\frac{1}{s}\sum_{j=1}^{N_{R}}{\left(\sum_{i=1}^{N_{G}}p_{RL}(i,j|\theta )\right)}^{2}=\frac{1}{s}\sum_{j=1}^{N_{R}}r^{2}(j|\theta )$$ (12)

Run Percentage (RP):

$$RP=\frac{1}{N}\sum_{i=1}^{N_{G}}\sum_{j=1}^{N_{R}}p_{RL}(i,j|\theta )=\frac{1}{N}\sum_{j=1}^{N_{R}}r(j|\theta )$$ (13)

Low Gray Level Run Emphasis (LGRE):

$$LGRE=\frac{1}{s}\sum_{i=1}^{N_{G}}\sum_{j=1}^{N_{R}}\frac{p_{RL}(i,j|\theta )}{i^{2}}$$ (14)

High Gray Level Run Emphasis (HGRE):

$$HGRE=\frac{1}{s}\sum_{i=1}^{N_{G}}\sum_{j=1}^{N_{R}}i^{2}p_{RL}(i,j|\theta )$$ (15)

Short Run Low Gray Level Emphasis (SRLGE):

$$SRLGE=\frac{1}{s}\sum_{i=1}^{N_{G}}\sum_{j=1}^{N_{R}}\frac{p_{RL}(i,j|\theta )}{i^{2}j^{2}}$$ (16)

Short Run High Gray Level Emphasis (SRHGE):

$$SRHGE=\frac{1}{s}\sum_{i=1}^{N_{G}}\sum_{j=1}^{N_{R}}\frac{i^{2}p_{RL}(i,j|\theta )}{j^{2}}$$ (17)

Long Run Low Gray Level Emphasis (LRLGE):

$$LRLGE=\frac{1}{s}\sum_{i=1}^{N_{G}}\sum_{j=1}^{N_{R}}\frac{j^{2}p_{RL}(i,j|\theta )}{i^{2}}$$ (18)

Long Run High Gray Level Emphasis (LRHGE):

$$LRHGE=\frac{1}{s}\sum_{i=1}^{N_{G}}\sum_{j=1}^{N_{R}}i^{2}j^{2}p_{RL}(i,j|\theta )$$ (19)

Gray Level Variance (GV):

$$GV=\sum_{i=1}^{N_{G}}\sum_{j=1}^{N_{R}}p_{RL}(i,j|\theta ){\left(i-\mu_{i}\right)}^{2}$$ (20)

Run-length Variance (RV):

$$RV=\sum_{i=1}^{N_{G}}\sum_{j=1}^{N_{R}}p_{RL}(i,j|\theta ){\left(j-\mu_{j}\right)}^{2}$$ (21)

Run-length Entropy (RE):

$$RE=-\sum_{i=1}^{N_{G}}\sum_{j=1}^{N_{R}}p_{RL}(i,j|\theta ){\text{log}}_{2}\left(p_{RL}(i,j|\theta )\right)$$ (22)

Texture measures were subsequently averaged over directions. SRE quantifies the distribution of short run-lengths, with greater value indicating the presence of shorter run-lengths in the parametric map which characterizes finer textures [60]. On the other hand, LRE measures the distribution of longer run-lengths, with greater values indicating the presence of longer run-lengths in the parametric image which represents coarser structural textures [60]. RLN assesses the similarity of run-lengths in the parametric image, with a lower value indicating more homogeneity among run-lengths in the image. RP Measures the coarseness of textures by taking the ratio of the number of runs with the total number of pixels in the image. SRE, LRE, RLN, and RP are typical features of run-length statistics [56,58]. However, these run-length features are defined by *r*(*j*|*θ*) the total number of runs of *j* pixels for all possible gray levels *i*, in the prescribed direction *θ*. Since for a given value of *r*(*j*|*θ*), the composition of runs can vary for different gray levels, features that depend solely on *r*(*j*|*θ*) would not be able to detect variation in gray levels [58]. In order to overcome this, LGRE and HGRE features were introduced [58]. LGRE and HGRE make use of the distribution of gray levels of runs [58]. LGRE measures the distribution of pixels with lower gray levels, with a greater value indicating a greater concentration of low gray levels in the parametric image. On the other hand, HGRE quantifies the distribution of pixels with higher gray levels, with a greater value indicating a greater concentration of high gray levels in the image. In a later study, features that measure joint distribution of run-length and gray levels were also introduced [59]. These include SRLGE, SRHGE, LRLGE, and LRHGE. SRLGE measures the joint distribution of shorter run-length with low gray levels. SRHGE quantifies the joint distribution of shorter run-length with high gray levels. LRLGE measures the joint distribution of longer run-length with low gray levels. LRHGE quantifies the joint distribution of longer run-length with high gray levels.

GV measures the variance of gray levels in the runs. RV is a measure of the variance in run for run-length. Run entropy (RE) quantifies the randomness in the distribution of run-lengths and gray levels. A higher value of RE indicates more texture randomness in the image.

#### GLSZM (Gray Level Size Zone Matrix) method

The GLSZM method quantifies texture by measuring the size of homogeneous zones for each gray level in an image [60,61]. A gray level zone is defined as an area of connected pixels with the same gray level. In the GLSZM matrix, *p*_*SZ*_(*i*, *j*) represents the number of gray level zones with gray level *i* and size *j* appearing in the image. In contrast to GLCM and GRLM methods, the GLSZM technique is direction independent. The computation of GLSZM matrix is based on run-length matrix calculation. From the GLSZM matrix, zone size features can be determined [60,61]. In the following equations, let $\mu_{i}=\sum_{i=1}^{N_{G}}\sum_{j=1}^{N_{S}}p_{SZ}(i,j)i$, $\mu_{j}=\sum_{i=1}^{N_{G}}\sum_{j=1}^{N_{S}}p_{SZ}(i,j)j$, and *N*_*S*_ is a dynamic number that represents the size of the largest flat zone in the image.

Small Area Emphasis (SAE):

$$SAE=\frac{1}{N_{Z}}\sum_{i=1}^{N_{G}}\sum_{j=1}^{N_{S}}\frac{p_{SZ}(i,j)}{j^{2}}$$ (23)

Large Area Emphasis (LAE):

$$LAE=\frac{1}{N_{Z}}\sum_{i=1}^{N_{G}}\sum_{j=1}^{N_{S}}j^{2}p_{SZ}(i,j)$$ (24)

Gray Level Nonuniformity (GLN):

$$GLN=\frac{1}{N_{Z}}\sum_{i=1}^{N_{G}}{\left(\sum_{j=1}^{N_{S}}p_{SZ}(i,j)\right)}^{2}$$ (25)

Size Zone Nonuniformity (SZN):

$$SZN=\frac{1}{N_{Z}}\sum_{j=1}^{N_{S}}{\left(\sum_{i=1}^{N_{G}}p_{SZ}(i,j)\right)}^{2}$$ (26)

Zone Percentage (ZP):

$$ZP=\frac{1}{N}\sum_{i=1}^{N_{G}}\sum_{j=1}^{N_{S}}p_{SZ}(i,j)$$ (27)

Low Gray Level Zone Emphasis (LGLZE):

$$LGLZE=\frac{1}{N_{Z}}\sum_{i=1}^{N_{G}}\sum_{j=1}^{N_{S}}\frac{p_{SZ}(i,j)}{i^{2}}$$ (28)

High Gray Level Zone Emphasis (HGLZE):

$$HGLZE=\frac{1}{N_{Z}}\sum_{i=1}^{N_{G}}\sum_{j=1}^{N_{S}}i^{2}p_{SZ}(i,j)$$ (29)

Small Area Low Gray Level Emphasis (SALGE):

$$SALGE=\frac{1}{N_{Z}}\sum_{i=1}^{N_{G}}\sum_{j=1}^{N_{S}}\frac{p_{SZ}(i,j)}{i^{2}j^{2}}$$ (30)

Small Area High Gray Level Emphasis (LAHGE):

$$SAHGE=\frac{1}{N_{Z}}\sum_{i=1}^{N_{G}}\sum_{j=1}^{N_{S}}\frac{i^{2}p_{SZ}(i,j)}{j^{2}}$$ (31)

Large Area Low Gray Level Emphasis (LALGE):

$$LALGE=\frac{1}{N_{Z}}\sum_{i=1}^{N_{G}}\sum_{j=1}^{N_{S}}\frac{j^{2}p_{SZ}(i,j)}{i^{2}}$$ (32)

Large Area High Gray Level Emphasis (LAHGE):

$$LAHGE=\frac{1}{N_{Z}}\sum_{i=1}^{N_{G}}\sum_{j=1}^{N_{S}}i^{2}j^{2}p_{SZ}(i,j)$$ (33)

Gray-level Variance (GLV):

$$GLV=\sum_{i=1}^{N_{G}}\sum_{j=1}^{N_{S}}p_{SZ}(i,j){\left(i-\mu_{i}\right)}^{2}$$ (34)

Zone Variance (ZV):

$$ZV=\sum_{i=1}^{N_{G}}\sum_{j=1}^{N_{S}}p_{SZ}(i,j){\left(j-\mu_{j}\right)}^{2}$$ (35)

Zone Entropy (ZE):

$$ZE=-\sum_{i=1}^{N_{G}}\sum_{j=1}^{N_{S}}p_{SZ}(i,j){\text{log}}_{2}\left(p(i,j)\right),$$ (36)

where $N_{Z}=\sum_{i=1}^{N_{G}}\sum_{j=1}^{N_{S}}p_{SZ}(i,j)$ is the total number of zones in the image.

SAE quantifies the distribution of small size zones. The greater value for SAE indicates that the image consists of more smaller size zones or finer textures. On the other hand, LAE measures the distribution of large area size zones. The greater value for LAE indicates an image with coarser textures. GLN quantifies the variability of gray level intensities in an image. A higher value for GLN indicates less homogeneity in the image. SZN quantifies the variability of size zones in the image with a higher value for SZN indicating less homogeneity in size zone areas. ZP quantifies the coarseness of the texture.

LGLZE measures the distribution of lower gray level size zones with higher values indicating a greater proportion of size zones distribution of low gray levels. HGLZE measures the distribution of higher gray level size zones. Higher values indicate a greater proportion of size zone distributions with high gray levels. SALGE estimates in the image the proportion of smaller size zones with lower gray levels. SAHGE measures the proportion of smaller size zones with higher gray levels in the image. LALGE measures the proportion of larger size zones with lower gray levels in an image whereas LAHGE estimates the proportion of larger size zones of higher gray levels in the image. Parameter GLV estimates the variance of gray levels for the zones whereas ZV measures the variance of size zones for the zones [60]. Parameter ZE assesses the randomness in the distribution of size zones and gray levels in the image. The higher values in ZE indicate more heterogeneity in the texture image.

### Classification algorithms

Mean-value parameters and textural features derived from GLCM, GRLM, and GLSZM methods were determined from each scan plane and averaged over all scan planes based on the ROI size. For each feature, we performed statistical analysis using MATLAB (Mathworks, Natick, Mass., USA) to check for any statistically significant difference between benign and malignant groups. To determine which tests to use, a Shapiro-Wilk normality test was performed on each feature to decide if it followed a normal distribution [27]. An unpaired *t-*test was used for a normally distributed feature. Otherwise, a non-parametric Mann-Whitney U-Test (two-sided, 95% confidence) was utilized. For these tests, *p*-values correction was not performed. The purpose of the statistical tests was solely to demonstrate the presence of discriminating features available for subsequent feature selection. These tests can gauge the resulting model performance as classification model developed using discriminating features will in general perform better compared to that developed using less discriminating features.

Using the GLCM method, a total of 25 mean-value and texture features were available for classification using either core or margin information. For the combined core and margin information, there were a total of 60 mean-value, texture, along with CMR and CMCR image quality features available for classification. Using the GRLM and GLSZM methods, a total of 75 mean-value and texture features were available for classification using either core or margin information. Combining both core and margin information, a total of 160 features were available for classification. These include mean-value, texture, along with CMR and CMCR image quality features.

The classification model was developed using the best combination of 10 features maximally. This was chosen based on the 10% rule of thumb to prevent overfitting [68]. Feature selection was performed using a forward sequential-feature-selection (SFS) that adds the feature one at a time up to a combination of 10 features. In each step, classification performance was evaluated. The selected features are those that provide the highest F1-Score (the harmonic average of precision and sensitivity) on the training set. We evaluated model performance using both LOOCV and hold-out validation. Leave-one-out cross-validation trains the model using all observations except one [27]. The process is repeated until all observations are left out for testing at least once [27]. The left-out observations are subsequently used for testing the developed model. As there are 193 observations in our cohort, this allows us to implement hold-out/split-sample validation. Hold-out validation will avoid performance over-estimation typically present using LOOCV [68]. This is appropriate to demonstrate the generalizability of the model to unseen testing sets. Hold-out validation randomly splits the data set into 70% training and 30% test sets. Model development was performed on the training set, while performance was evaluated on the unseen testing set. To account for the random partitioning process, several realizations were evaluated. The classification performance was found through averaging the results over ten different realizations of the data.

Standard classification algorithms were utilized and implemented using a custom software in MATLAB. These included: LDA, KNN, and nonlinear classifier in SVM-RBF. In addition, we also implemented an ANN using neural network pattern recognition tool (nprtool) in MATLAB. The performance of these classification algorithms was assessed using the ROC analysis utilizing sensitivity, specificity, accuracy, AUC, PPV, and NPV metrics. Using probabilistic generative models, LDA can be described as estimating the posterior probability of assigning an input vector **x** into one of the two classes by assuming that probability density function of each class is a Gaussian [69]. A KNN is an instance-based learning algorithm that predicts class association of a test point in the feature space based on the majority of the points neighbouring the test point and the distance between those points to the test point. The KNN classifier used *k* = 1, 3, 5 nearest neighbours. The SVM-RBF creates a model that maximizes the margin between the two classes and predicts class association of the testing data based on which side of the gap they fall on [70]. RBF kernel is used to map the input data into a higher-dimensional space where the data are supposed to have better distribution, prior to selecting an optimal separating hyperplane in this higher-dimensional feature space. The soft margin parameter C and free parameter γ are the parameters of the kernel. These parameters were optimized using a grid search method.

Implementation of ANN using nprtool randomly partitions the data into training, validation, and testing subsets with 70%, 15%, and 15% proportion in each subset. The process is also repeated ten times, and classification performances were averaged over different partition realizations. The developed network is a shallow two-layer feedforward network that consists of a single hidden layer and an output layer. The network uses a sigmoid transfer function in the hidden layer and a soft-max transfer function in the output layer. The number of neurons in the hidden layer was set to 20. Training of the network involves updating weight and bias values to optimize the network’s performance. The default performance function for feedforward network is the mean square error: averaged square error between the network output and the target output. Training was implemented in batch mode using an algorithm that updates the weight and bias values according to Levenberg-Marquardt optimization (trainlm function in MATLAB). The batch size was 193. In Levenberg-Marquardt algorithm, the Jacobian of the performance with respect to weight and bias variables was calculated using backpropagation algorithm. The default trainlm training parameters were used. These include: the maximum number of epochs to train = 1000, performance goal = 0, minimum gradient = 1e-7, maximum validation failures = 6, initial adaptive value mu = 0.001, mu decrease factor = 0.1, mu increase factor = 10, and maximum mu = 1000. Validation sets were used to suspend training early, if the performance on validation errors fails to improve or remains the same for 6 times in a row. Testing sets were used to further check for the generalizability of the network.

## Results

**Fig 1:**
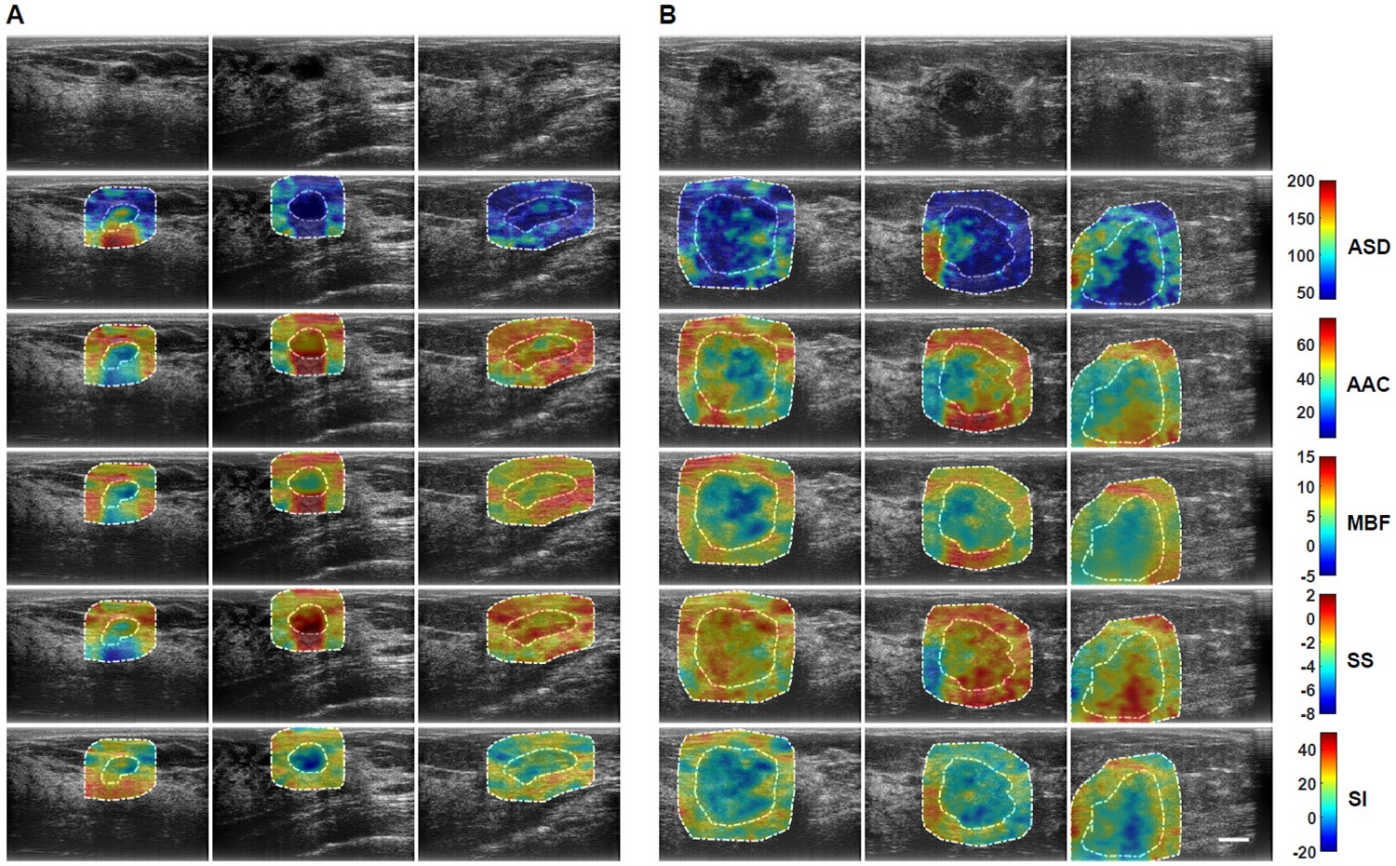
Representative B-mode and QUS spectral parametric images of ASD, AAC, MBF, SS, and SI from A benign (left three columns) and B malignant (right three columns) breast lesions. The colour-bar range is 160 μm for ASD, 70 dB/cm^3^ for AAC, 20 dB for MBF, 10 dB/MHz for SS, and 70 dB for SI. The scale bar represents 1cm. The benign breast lesions were diagnosed as fibroadenomas, and complicated a cyst, respectively. The malignant lesions were diagnosed as invasive ductal carcinomas (IDC), invasive mammary carcinoma, and invasive lobular carcinoma (ILC). Using these parametric images, mean-value, textural, and image quality features were determined as imaging biomarkers for the characterization of breast lesions.

US RF data were acquired from 193 patients in this study. Patients were aged 20 to 89 and 92 patients had benign masses and 101 patients had malignant masses in the research group. Patient and breast mass characteristics are provided in S1 and S2 Tables. In addition, Breast Imaging Reporting and Data Systems (BI-RADS) distribution among the lesions is also presented in S3 Table. Fig 1 presents representative B-mode US and parametric images of ASD, AAC, MBF, SS, and SI from both benign and malignant groups. The benign lesions in this study were diagnosed as predominantly fibroadenomas (n = 46) and cysts/complicated cysts (n = 21). The malignant lesions were diagnosed as invasive ductal carcinoma (IDC) (n = 80) and invasive mammary carcinoma (n = 7), respectively. Mean-value parameters were determined as well as GLCM, GRLM, and GLSZM texture parameters, along with image quality features from these parametric images and were evaluated based on their performance as imaging biomarkers associated with discriminating between benign and malignant lesions. Sonographically, benign lesions demonstrated better defined borders and appeared overall less spiculated. In the parametric images, benign lesions demonstrated less obvious heterogeneity than was apparent in malignant lesions.

**Fig 2:**
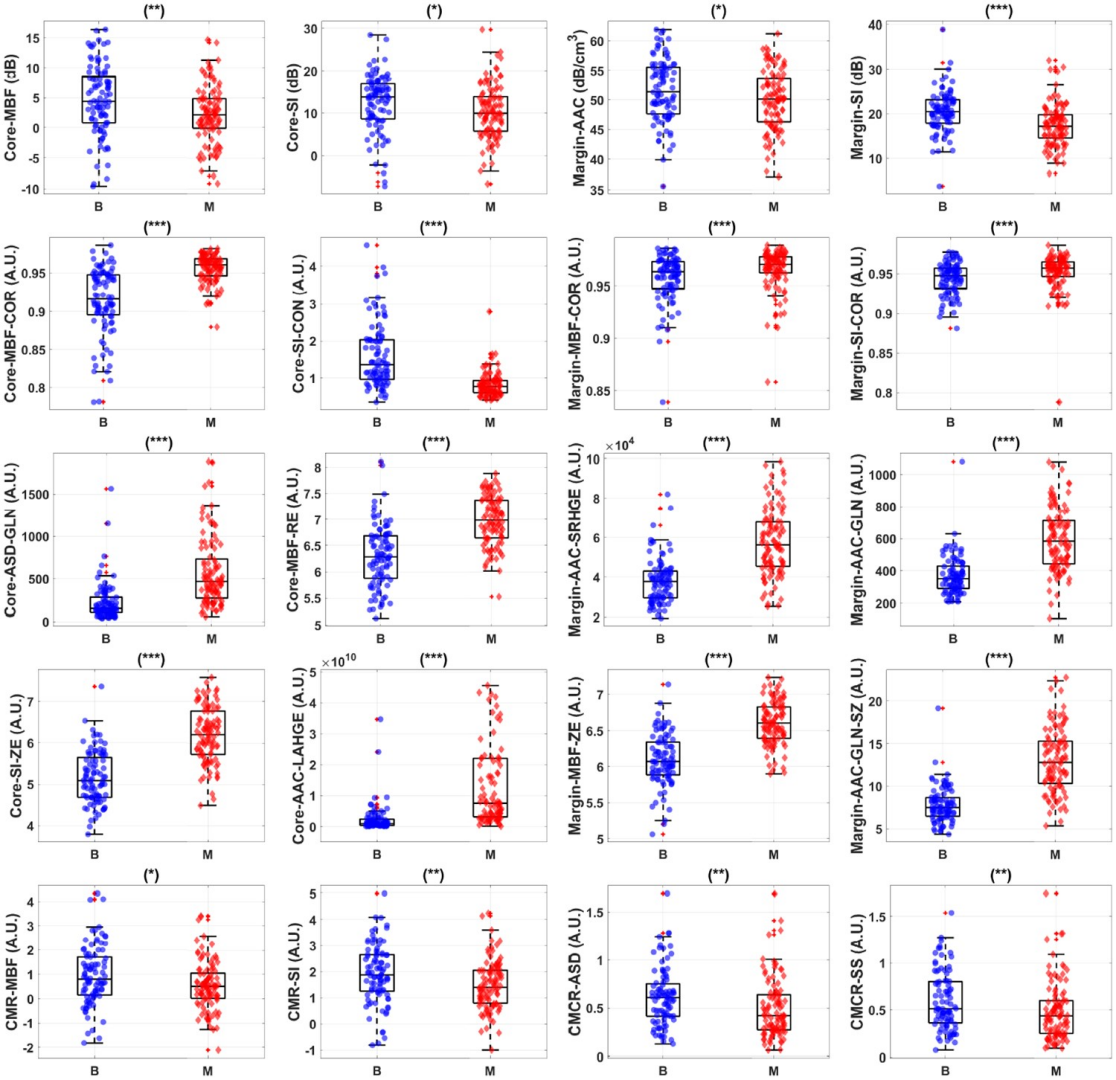
Representative box and scatter plots of features that demonstrate statistically significant difference (*p*-values *<* 0.05) between benign (‘B’) and malignant (‘M’) lesion groups. The first row shows core and margin mean-value parameters. The second row depicts representative core and margin GLCM features that showed discriminative power. The third row shows representative core and margin GRLM features that discriminate the two lesion groups. The last row depicts representative GLSZM features that provided the most discriminative power. There is a total of 160 features from tumour core and 5-mm margin, including 10 image quality features, available for feature selection. Among these features, 4 mean-values, 125 textural, and 1 image quality features demonstrate statistically significant difference between the two lesions. Statistically significant (*p <* 0.05), highly significant (*p <* 0.01), and extremely significant (*p <* 0.001) are shown with (*), (**), and (***), respectively.

Fig 2 shows representative box and scatter plots of mean-value, GLCM, GRLM, and GLSZM texture values, along with image quality features that demonstrated statistically significant differences (*p* < 0.05) between benign and malignant breast lesions. Six mean-value, 24 GLCM, 127 GRLM, 126 GLSZM texture, and 4 image quality features demonstrated statistically significant differences (*p* < 0.05). Features were further subclassified based on their degree of statistical significance. Statistically significant (*p* < 0.05), highly significant (*p* < 0.01), and extremely significant (*p* < 0.001) features are indicated with (*), (**), and (***), respectively. Among the mean-value parameters from the core, MBF, SI, and AAC demonstrated statistically significant differences *(p* < 0.05). The MBF, SI, and AAC parameters from the core were 4.4 ± 0.6 dB versus 2.2 ± 0.5 dB, 13.8 ± 0.7 dB versus 10.0 ± 0.6 dB, and 46.9 ± 0.9 dB/cm^3^ versus 43.6 ± 0.7 dB/cm^3^ for benign and malignant lesions, respectively. Among the mean-value parameters from the margin, the MBF, SI, and AAC also demonstrated statistically significant differences (*p* < 0.05). The MBF, SI, and AAC from the margin are 11.2 ± 0.3dB versus 9.2 ± 0.3dB, 20.4 ± 0.5dB versus 17.1 ± 0.5dB, and 51.4 ± 0.6dB/cm^3^ versus 50.1 ± 0.5dB/cm^3^ for benign and malignant lesions, respectively.

Table 1 lists an optimum set of features from GLCM, GRLM, and GLSZM methods that contributed to a hybrid biomarker that best separated benign from malignant lesions using breast mass core and margin information. The best classification performance using the SVM-RBF was achieved using features derived from the GLSZM methodology: Margin-MBF-GLN-SZ, Margin-SI-GLN-SZ, Margin-MBF-LGZE, Core-MBF-GV-SZ, Core-SS-GLN-SZ, Margin-AAC-GV-SZ, Core-SS-LGZE, Margin-MBF-SALGE, Margin-SS-SZN, and Core-ASD-SAE. Texture features dominated the best hybrid biomarker which separated patients into whether they had benign or malignant characteristics.

**Table 1:** Optimum feature set for classification using both core and margin information utilizing GLCM, GRLM, and GLSZM texture methods and SVM-RBF classification algorithm. A maximum of 10 features was selected for classification. Model performance was evaluated using LOOCV method. Features were selected using forward SFS based on F1-score metric. Textural features, for example Core-MBF-CON: GLCM contrast parameter of MBF parametric image from core ROI and Margin-MBF-SALGE: GLSZM small area low gray level emphasis parameter of MBF parametric image from margin ROI, were the dominant features that contributed to hybrid biomarkers that best separated the two lesion types.

GLCM Selected Features	GRLM Selected Features	GLSZM Selected Features
Core-MBF-CON	Core-MBF-SRE	Margin-MBF-GLN-SZ
Margin-MBF	CMCR-MBF	Margin-SI-GLN-SZ
CMR-AAC	Margin-AAC-SRE	Margin-MBF-LGZE
Margin-SS	Margin-MBF-RP	Core-MBF-GV-SZ
CMCR-AAC	Margin-AAC-RP	Core-SS-GLN-SZ
Margin-AAC	CMCR-AAC	Margin-AAC-GV-SZ
Core-SS-CON	Core-AAC-SRE	Core-SS-LGZE
Margin-ASD-CON	Core-MBF-RP	Margin-MBF-SALGE
Core-ASD-CON	Core-AAC-RP	Margin-SS-SZN
CMR-SS	Margin-SI-RP	Core-ASD-SAE

Table 2A and 2B tabulate classification performance utilizing core GLCM features evaluated using LOOCV and hold-out validation, respectively. Using LOOCV, the SVM-RBF provided the best classification performance of 84% sensitivity, 78% specificity, 81% accuracy, 0.88 AUC, 81% PPV, and 82% NPV. Using hold-out validation, the ANN resulted in the best performance of 89% sensitivity, 77% specificity, 83% accuracy, 0.92 AUC, 81% PPV, and 86% NPV.

**Table 2:** A: Core classification results of GLCM methodology using LOOCV. B: Core classification results of GLCM methodology using hold-out validation.

Classifier	Sensitivity	Specificity	Accuracy	AUC	PPV	NPV
**LDA**	86%	73%	80%	0.84	78%	83%
**KNN**	75%	71%	73%	0.77	74%	72%
**SVM-RBF**	84%	78%	81%	0.88	81%	82%
**LDA**	79%	65%	72%	0.81	71%	74%
**KNN**	72%	67%	70%	0.77	71%	69%
**SVM-RBF**	87%	69%	79%	0.82	76%	83%
**ANN**	89%	77%	83%	0.92	81%	86%

Table 3A and 3B show classification performance utilizing margin GLCM features evaluated using LOOCV and hold-out validation, respectively. Using LOOCV, the SVM-RBF attained the best performance of 81% sensitivity, 75% specificity, 78% accuracy, 0.80 AUC, 78% PPV, and 78% NPV. Using hold-out validation technique, the ANN resulted in the best classification performance of 70% sensitivity, 80% specificity, 75% accuracy, 0.84 AUC, 81% PPV, and 72% NPV.

**Table 3:** A: Margin classification results of GLCM methodology using LOOCV. B: Margin classification results of GLCM methodology using hold-out validation.

Classifier	Sensitivity	Specificity	Accuracy	AUC	PPV	NPV
**LDA**	68%	74%	71%	0.75	74%	68%
**KNN**	75%	66%	71%	0.76	71%	71%
**SVM-RBF**	81%	75%	78%	0.80	78%	78%
**LDA**	60%	71%	65%	0.73	70%	61%
**KNN**	61%	60%	61%	0.61	64%	58%
**SVM-RBF**	67%	65%	66%	0.69	68%	64%
**ANN**	70%	80%	75%	0.84	81%	72%

Table 4A and 4B present classification performance utilizing core and margin GLCM features evaluated using LOOCV and hold-out validation, respectively. Using LOOCV, the SVM-RBF provided the best classification performance of 86% sensitivity, 83% specificity, 84% accuracy, 0.90 AUC, 84% PPV, and 84% NPV. Using hold-out validation, the ANN resulted in the best classification performance of 88% sensitivity, 78% specificity, 83% accuracy, 0.92 AUC, 83% PPV, and 87% NPV. Core GLCM features alone resulted in the highest 89% sensitivity detection of malignancy and overall accuracy and AUC of 83% and 0.92, respectively.

**Table 4:** A: Core and margin classification results of GLCM methodology using LOOCV. B: Core and margin classification results of GLCM methodology using hold-out validation.

Classifier	Sensitivity	Specificity	Accuracy	AUC	PPV	NPV
**LDA**	83%	78%	81%	0.86	81%	81%
**KNN**	82%	72%	77%	0.84	76%	79%
**SVM-RBF**	86%	83%	84%	0.90	84%	84%
**LDA**	74%	67%	71%	0.80	72%	71%
**KNN**	72%	67%	69%	0.74	71%	68%
**SVM-RBF**	78%	64%	71%	0.81	72%	73%
**ANN**	88%	78%	83%	0.92	83%	87%

Table 5A and 5B tabulate classification performance utilizing core GRLM features evaluated using LOOCV and hold-out validation, respectively. Using LOOCV, the SVM-RBF provided the best classification performance of 90% sensitivity, 83% specificity, 87% accuracy, 0.87 AUC, 85% PPV, 88% NPV. Using hold-out validation, the ANN resulted in the best classification performance of 86% sensitivity, 82% specificity, 84% accuracy, 0.93 AUC, 84% PPV, and 86% NPV.

**Table 5:** A: Core classification results of GRLM methodology using LOOCV. B: Core classification results of GRLM methodology using hold-out validation.

Classifier	Sensitivity	Specificity	Accuracy	AUC	PPV	NPV
**LDA**	85%	76%	81%	0.87	80%	82%
**KNN**	85%	76%	81%	0.84	80%	82%
**SVM-RBF**	90%	83%	87%	0.87	85%	88%
**LDA**	69%	77%	73%	0.82	78%	70%
**KNN**	70%	67%	68%	0.74	70%	67%
**SVM-RBF**	72%	71%	72%	0.75	74%	70%
**ANN**	86%	82%	84%	0.93	84%	86%

Table 6A and 6B tabulate classification performance utilizing margin GRLM features evaluated using LOOCV and hold-out validation, respectively. Using LOOCV, the SVM-RBF resulted in the best classification performance of 85% sensitivity, 86% specificity, 85% accuracy, 0.87 AUC, 87% PPV, 84% NPV. Using hold-out validation, the ANN obtained the best classification performance of 90% sensitivity, 84% specificity, 87% accuracy, 0.93 AUC, 87% PPV, 88% NPV.

**Table 6:** A: Margin classification results of GRLM methodology using LOOCV. B: Margin classification results of GRLM methodology using hold-out validation.

Classifier	Sensitivity	Specificity	Accuracy	AUC	PPV	NPV
**LDA**	74%	86%	80%	0.82	85%	75%
**KNN**	85%	83%	84%	0.82	84%	84%
**SVM-RBF**	85%	86%	85%	0.87	87%	84%
**LDA**	65%	85%	74%	0.83	83%	69%
**KNN**	72%	78%	75%	0.79	80%	72%
**SVM-RBF**	73%	67%	70%	0.76	72%	70%
**ANN**	90%	84%	87%	0.93	87%	88%

Table 7A and 7B tabulate classification performance utilizing core and margin GRLM features evaluated using LOOCV and hold-out validation, respectively. Using LOOCV, the SVM-RBF achieved the best classification performance of 86% sensitivity, 85% specificity, 85% accuracy, 0.87 AUC, 86% PPV, 85% NPV. Using hold-out validation, the ANN achieved the best classification performance of 92% sensitivity, 86% specificity, 89% accuracy, 0.95 AUC, 88% PPV, 90% NPV. Margin GRLM features performed better than core GRLM features. Combining features from both the core and the margin resulted in improved classification performance with 92% sensitivity and accuracy and AUC of 89% and 0.95, respectively.

**Table 7:** A: Core and margin classification results of GRLM methodology using LOOCV. B: Core and margin classification results of GRLM methodology using hold-out validation.

Classifier	Sensitivity	Specificity	Accuracy	AUC	PPV	NPV
**LDA**	77%	85%	81%	0.82	85%	77%
**KNN**	86%	85%	85%	0.83	86%	85%
**SVM-RBF**	86%	85%	85%	0.87	86%	85%
**LDA**	70%	81%	75%	0.84	81%	72%
**KNN**	74%	77%	75%	0.79	79%	73%
**SVM-RBF**	71%	74%	72%	0.77	77%	70%
**ANN**	92%	86%	89%	0.95	88%	90%

Table 8A and 8B tabulate classification performance using core GLSZM features evaluated using LOOCV and hold-out validation, respectively. Using LOOCV, the SVM-RBF resulted in the best classification performance of 90% sensitivity, 85% specificity, 88% accuracy, 0.89 AUC, 87% PPV, 89%. Using hold-out validation, the ANN achieved the best performance of 93% sensitivity, 88% specificity, 91% accuracy, 0.95 AUC, 90% PPV, 92% NPV.

**Table 8:** A: Core classification results of GLSZM methodology using LOOCV. B: Core classification results of GLSZM methodology using hold-out validation.

Classifier	Sensitivity	Specificity	Accuracy	AUC	PPV	NPV
**LDA**	82%	87%	84%	0.87	87%	82%
**KNN**	84%	80%	82%	0.82	83%	82%
**SVM-RBF**	90%	85%	88%	0.89	87%	89%
**LDA**	77%	76%	77%	0.85	79%	75%
**KNN**	75%	74%	75%	0.78	77%	73%
**SVM-RBF**	75%	72%	74%	0.80	75%	72%
**ANN**	93%	88%	91%	0.95	90%	92%

Table 9A and 9B tabulate the classification performance utilizing margin GLSZM features using LOOCV and hold-out validation, respectively. Using LOOCV, the SVM-RBF resulted in the best classification performance of 90% sensitivity, 90% specificity, 90% accuracy, 0.91 AUC, 91% PPV, 89% NPV. Using hold-out validation, the ANN provided the best performance of 89% sensitivity, 91% specificity, 90% accuracy, 0.95 AUC, 92% PPV, 88% NPV.

**Table 9:** A: Margin classification results of GLSZM methodology using LOOCV. B: Margin classification results of GLSZM methodology using hold-out validation.

Classifier	Sensitivity	Specificity	Accuracy	AUC	PPV	NPV
**LDA**	84%	89%	87%	0.89	89%	84%
**KNN**	87%	85%	86%	0.90	86%	86%
**SVM-RBF**	90%	90%	90%	0.91	91%	89%
**LDA**	69%	87%	78%	0.88	87%	72%
**KNN**	75%	79%	77%	0.81	81%	75%
**SVM-RBF**	74%	86%	80%	0.88	87%	76%
**ANN**	89%	91%	90%	0.95	92%	88%

Table 10A and 10B tabulate classification performance utilizing core and margin GLSZM features evaluated using LOOCV and hold-out validation. Using LOOCV, the SVM-RBF resulted in the best classification performance of 90% sensitivity, 90% specificity, 90% accuracy, 0.90 AUC, 91% PPV, 89% NPV. Using hold-out validation, the ANN achieved the best performance of 89% sensitivity, 91% specificity, 90% accuracy, 0.96 AUC, 92% PPV, 89% NPV.

**Table 10:** A: Core and margin classification results of GLSZM methodology using LOOCV. B: Core and margin classification results of GLSZM methodology using hold-out validation.

Classifier	Sensitivity	Specificity	Accuracy	AUC	PPV	NPV
**LDA**	80%	93%	87%	0.87	93%	81%
**KNN**	85%	87%	86%	0.87	88%	84%
**SVM-RBF**	90%	90%	90%	0.90	91%	89%
**LDA**	71%	90%	80%	0.87	90%	74%
**KNN**	72%	80%	76%	0.80	81%	72%
**SVM-RBF**	80%	84%	82%	0.87	86%	79%
**ANN**	89%	91%	90%	0.96	92%	89%

These results suggested that GRLM and GLSZM features outperform those of GLCM features. This supports our hypothesis that one texture analysis method may perform better than the others. Using LOOCV, our results indicated that core classification performs better than margin classification in the case of GLCM and GRLM. For the GLSZM, there was a slight improvement in performance by combining core and rim features, compared to that using core features alone. Using hold-out validation, core classification performed better than margin classification using GLCM features. However, using GRLM or GLSZM features to develop a model, resulted in better margin classification than core classification. Overall, combining both core and margin information resulted in improved classification performance. Among the validation techniques, LOOCV led to better performance than hold-out validation. Although decreases in classification performance were observed using the latter validation method, the best averaged performance of 91% accuracy and 0.95 AUC were obtained utilizing GLSZM features from the tumour core, using the ANN. GLSZM features also attained 90% accuracy and 0.96 AUC with core and margin information using the ANN. Advanced machine learning classifier in ANN proves to be more robust in generalizing diagnostic model inference, compared to those of standard classifiers.

## Discussion

In this study, the performance of different texture analysis methods applied on QUS spectral parametric images for the characterization of breast lesions was demonstrated, for the first time. Textural features derived from the GLCM, GRLM, and GLSZM methods were used as imaging biomarkers to develop a diagnostic model for classifying breast lesions as either benign or malignant. In addition to analyzing features from tumour core, analyses conducted also included peri-tumoural tissue (5-mm margin extending from tumour core). In invasive tumours, the rim contains infiltrating components that extend from tumour core into the surrounding tissue [71]. Earlier, tumour rim analysis has been used to predict the response to NAC [62]. Here, rim analysis was used to characterize breast lesions. This study builds upon previous studies through a significant expansion of the cohort and a comparison of different texture methods. Earlier, the cohort consisted of 78 patients with breast lesions (46 benign and 32 malignant cases) [8]. Recently, novel derivative texture methods were also evaluated on a larger cohort of patients with breast lesions [9]. In those studies, however, only the GLCM method was used to quantify texture of the parametric images. In the current study, different texture methods were applied here on a larger cohort of 193 patients with breast lesions (92 benign and 101 malignant cases). Larger cohort allowed assessment of the model performance using both LOOCV and hold-out validation. The latter demonstrates model generalizability to independent testing sets. Findings from this study suggest that different texture methods can affect classification performance. Specifically, tumour core features derived from the GLSZM method demonstrated the best classification performance of 93% sensitivity, 88% specificity, 91% accuracy, 0.95 AUC, 90% PPV, and 92% NPV with hold-out validation, utilizing the ANN.

In a previous study, the average-values of MBF, SI, and AAC images did not show statistically significant differences (*p* < 0.05) [8]. In the study here, however, these parameters from tumour core and a 5-mm margin showed statistically significant differences (*p* < 0.05). This can be attributed to the increased size of the cohort. The same trend was also observed in our recent study [9]. Malignant lesions exhibited lower MBF, SI, and AAC compared to those of benign lesions. An earlier study also observed the same trend of lower QUS spectral parameters in cancerous versus those of normal breast tissues [38]. Furthermore, this observation is also generally consistent with sonographic features of B-mode US images of breast nodules where marked hypo-echogenicity was observed in the malignant lesions compared to the benign lesions [10]. The MBF and SI represent tissue microstructural characteristics that include the size, shape, number, and organization of acoustic scatterers, along with their elastic properties [19]. On the other hand, the AAC reflects scatterers number density, organization, and their elastic properties [19]. Histopathological analysis has demonstrated that related tissue structural properties are distinct between benign and malignant lesions [72]. A more regular arrangement of cells is observed in benign lesions [72]. In contrast, malignant lesions exhibit cellularity-rich areas with a tendency to form cell clusters [72].

As average-based parameters do not preserve information regarding tumour heterogeneity, texture analysis is needed. Texture analysis of QUS spectral parametric images can quantify lesion heterogeneities that includes variations in size, density, and distribution of acoustic scatterers. Better discrimination of different histological tissue types is potentially achievable using these imaging biomarkers compared to mean-value parameters. Among the GLCM features, 24 biomarkers showed statistically significant differences (*p* < 0.05). Among the GRLM features, 127 biomarkers demonstrated statistically significant differences (*p* < 0.05). Among the GLSZM features, 126 biomarkers showed statistically significant differences (*p* < 0.05). In addition, QUS spectral and texture analyses of tumour core and its 5-mm margin allowed us to obtain image quality features including the CMR and CMCR. Here, the CMR of MBF and SI demonstrated statistically significant differences (*p* < 0.05). Additionally, the CMCR of ASD and SS also showed statistically significant differences (*p* < 0.05).

Previously, evaluation of mean-value parameters and GLCM texture features for breast lesion characterization on a smaller subset of 78 patients achieved the best classification performance of 96% sensitivity, 84% specificity, 91% accuracy and 0.97 AUC [8]. However, application of the same approach on a larger cohort of 193 patients was only able to achieve the best classification performance of 84% sensitivity, 78% specificity, 81% accuracy, and 0.88 AUC, as shown in Table 2A. This suggests that generalization of GLCM-based texture analysis for breast lesions characterization is not optimum. However, when different texture methods were considered, results like that of a recent study that include mean-value parameters, GLCM texture, and novel GLCM texture-derivate features of QUS spectral parametric images were achieved [9]. Past studies on classification using different texture methods suggested that GLCM-based features performed the least optimum in comparison to those using run-length (GRLM) and size zone (GLSZM) features [61]. This is consistent with the observations in the study here where GLSZM proved to be the optimum texture analysis approach for breast lesion characterization (91% accuracy and 0.95 AUC using core analysis and the ANN). The GRLM method marginally underperformed the GLSZM method (89% accuracy vs 91% accuracy). This is not unexpected as both techniques have similar matrix construction, albeit different interpretation. The results suggest that optimizing methods for extracting discriminative textural features can improve classification performance. Further application of derivative texture methods using GLSZM texture analysis on the QUS spectral parametric images can potentially improve the classification performance further. This type of investigation will be conducted in future studies.

Here, we evaluated model performance using LOOCV and hold-out validation. As expected, LOOCV led to better classification results than those of hold-out validation. However, hold-out validation is necessary to demonstrate the generalizability of the model. In terms of the classification algorithms, nonlinear classifiers in the SVM-RBF and ANN proved to be more robust to random data partitioning compared to the LDA and KNN, allowing for better generalization. Using LOOCV, the best classification performance of 90% sensitivity, 90% specificity, 90% accuracy, 0.91 AUC, 91% PPV, and 89% NPV was achieved, utilizing margin GLSZM texture features and the SVM-RBF. Using hold-out validation, core GLSZM features resulted in the best average performance of 93% sensitivity, 88% specificity, 91% accuracy, 0.95 AUC, 90% PPV, and 92% NPV using the ANN. Although random partitioning of the data can result in sub-optimal classification performance, the network was still able to pick up necessary patterns in the training data and generalize in predicting class association of the testing set. As more breast US RF data from patients are acquired over time, ANN and deep learning techniques may prove to be more effective classification algorithms for maximizing the classification performance.

Past studies have demonstrated the use of B-mode US images and texture analysis of these images in the characterization of breast lesions. Stavros *et al*. performed manual classification of solid breast nodules in 750 patients (625 benign and 125 malignant) using B-mode US images [10]. Using sonographical features of the lesions (for example echogenicity, shape, contour, and surrounding tissue), they achieved 98% sensitivity, 68% specificity, and 73% accuracy [10]. Tsui *et al*. analyzed the statistics of backscattered echo envelope using Nakagami statistical model, attaining 92% sensitivity, 72% specificity, and 82% accuracy in the characterization of 100 patients with breast tumours (50 benign and 50 malignant) [73]. Furthermore, Destrempes *et al*. explored various combinations of features from shear wave elastography (SWE), RF spectral analysis, and echo envelope statistical analysis, along with BI-RADS score in the classification of 103 suspicious solid breast lesions from 103 patients (BI-RADS 3–4) [74]. They observed that the combination of SWE, QUS, and BI-RADS scoring led to an AUC of 0.97, with 76% specificity at 98% sensitivity [74]. In addition, Dobruch-Sobczak *et al*. also found that the combination of echo envelope statistics features and BI-RADS scoring achieved 100% sensitivity, 55% specificity, and an AUC of 0.97 in the classification of 107 solid or cystic-solid breast lesions from 78 patients [75]. Gomez *et al*. utilized GLCM textural features from 436 breast US images (219 benign and 217 carcinoma) in the characterization of breast lesions, achieving classification performance of 70% sensitivity, 77% specificity, and 74% accuracy [54]. Further works involving the applications of ANN on breast US images resulted in an improved classification performance with 92% sensitivity, 91% specificity, and 91% accuracy [11,12]. Recently, Han *et al*. utilized a deep learning framework to differentiate benign from malignant breast lesions using the GoogLeNet convolutional neural network (CNN) on a large data set comprised of 7,408 breast US images from 5,151 patients [13]. They obtained 90% accuracy, 86% sensitivity, 96% specificity, and 0.90 AUC [13]. In another study, Byra *et al*. also developed a deep learning-based approach using deep CNN to classify breast lesions on 882 breasts US images [14]. The trained network achieved 0.94 AUC on the test set of 150 cases [14]. Recently, Osapoetra *et al*. performed characterization of breast lesions using the combination of several single biomarkers from mean-value parameters, texture, and texture-derivate features of QUS spectral parametric images, achieving 90% sensitivity, 92% specificity, and 91% accuracy, and 0.93 AUC using the SVM-RBF classifier [9]. In that study, the GLCM method was used to extract texture and texture-derivate features from tumour core and its 5-mm margin. In this study, implementation of hold-out validation for assessing model performance resulted in the best classification performance of 93% sensitivity, 88% specificity, 91% accuracy, and 0.95 AUC using mean-value parameters, GLSZM texture, and image quality features. This demonstrates the generalizability of the QUS spectroscopy framework and texture methods in the characterization of breast lesions. Our results suggest that different methods for extracting textural features of QUS spectral parametric images can result in the same classification performance to that obtained using more computationally intensive derivative texture methods [9].

## Conclusion

QUS-based techniques, along with optimized texture methods, provided an improved classification performance for the characterization of breast lesions compared to past work utilizing sonographical features of B-mode US images and other RF-based work. This result can be attributed to the fact that QUS techniques measure independent intrinsic acoustic and mechanical properties of tissue microstructure that are distinct between benign and more structurally disorganized malignant lesions. In addition, QUS spectral analysis allows measurement of instrument- and operator-independent tissue properties through a normalization procedure. In the work here, the classification of breast lesions using these imaging biomarkers obtained from different texture methods resulted in a more robust classification model. Evaluation of QUS spectroscopy and texture analysis methods in a larger cohort using proper validation technique demonstrate the generalization of the proposed framework. Furthermore, QUS spectroscopy does not use ionizing radiation and does not need the administration of exogenous contrast agents. These advantages of QUS spectroscopy, along with texture analyses, over other imaging modalities including x-ray mammography, standard B-mode US, and contrast-enhanced MRI make it an ideal tool for rapid and accurate breast cancer diagnosis in clinical settings.

## Supporting information

S1 Table Benign patient characteristics. Lesion size refers to the longest dimension of the tumor.
(DOCX)

S2 Table Malignant patient characteristics. Lesion size refers to the longest dimension of the tumor. ER is estrogen receptor, PR is progesterone receptor, HER2 is human epithelial growth factor receptor 2. Lesion size refers to the longest dimension of the tumor. IDC stands for invasive ductal carcinoma. ILC stands for invasive lobular carcinoma. DCIS stands for ductal carcinoma *in situ*, IMC stands for invasive mammary carcinoma.
(DOCX)

S3 Table BI-RADS Distribution among the lesions evaluated. (DOCX)
