# Supplementary material for: Comparison of methods for texture analysis of QUS parametric images in the characterization of breast lesions
Source: PLoS One. 2020 Dec 31;15(12):e0244965. doi: 10.1371/journal.pone.0244965 (PMC7775053; doi:10.1371/journal.pone.0244965)
Supplement: S1 Table — Lesion size refers to the longest dimension of the tumor. (DOCX) [file pone.0244965.s001.docx]

**S1 Table: Benign Patient Characteristics.** Lesion size refers to the longest dimension of the tumor.

| **Patient Number** | **Age** | **Type** | **Size (cm)** |
| --- | --- | --- | --- |
| **1** | **46** | **Fibroadenoma** | **1.8** |
| **2** | **39** | **Fibroadenoma** | **1.1** |
| **3** | **43** | **Fibroadenoma** | **1.0** |
| **4** | **40** | **Lactating Adenoma** | **2.3** |
| **5** | **21** | **Cyst** | **0.8** |
| **6** | **42** | **Fibroadenomatoid Changes** | **1.2** |
| **7** | **52** | **Cyst** | **2.6** |
| **8** | **34** | **Fibroadenoma** | **0.8** |
| **9** | **39** | **Benign Nodule** | **1.2** |
| **10** | **62** | **Fibroadenoma** | **1.1** |
| **11** | **58** | **Seroma** | **5.6** |
| **12** | **39** | **Fibroadenoma** | **1.1** |
| **13** | **51** | **Fibroadenoma** | **2.3** |
| **14** | **60** | **Cyst** | **0.6** |
| **15** | **54** | **Fibroadenoma** | **1.0** |
| **16** | **67** | **Cyst** | **0.8** |
| **17** | **53** | **Cyst** | **0.6** |
| **18** | **31** | **Fibroadenoma** | **1.3** |
| **19** | **44** | **Fibroadenoma** | **1.5** |
| **20** | **66** | **Cyst** | **0.7** |
| **21** | **45** | **Fibroadenoma** | **0.4** |
| **22** | **33** | **Fibroadenoma** | **1.3** |
| **23** | **43** | **Adenosis & Fibrosis** | **1.0** |
| **24** | **44** | **Cyst** | **0.8** |
| **25** | **50** | **Fibroadenoma** | **0.3** |
| **26** | **27** | **Fibroadenoma** | **1.4** |
| **27** | **38** | **Fibroadenoma** | **0.7** |
| **28** | **45** | **Cyst** | **0.7** |
| **29** | **62** | **Fibroadenoma** | **1.5** |
| **30** | **72** | **Cyst** | **0.5** |
| **31** | **53** | **Benign Nodule** | **3.1** |
| **32** | **46** | **Fibroadenoma** | **2.9** |
| **33** | **63** | **Apocrine Metaplasia & Usual Ductal Hyperplasia** | **0.9** |
| **34** | **38** | **Fibroadenoma** | **1.9** |
| **35** | **31** | **Fibroadenoma** | **2.7** |
| **36** | **49** | **Fibroadenoma** | **1.0** |
| **37** | **39** | **Fibroadenoma** | **1.2** |
| **38** | **57** | **Benign Nodule** | **0.9** |
| **39** | **61** | **Fibroadenoma** | **1.8** |
| **40** | **57** | **Fibrosis** | **1.6** |
| **41** | **41** | **Fibroadenoma** | **2.7** |
| **42** | **33** | **Scar/Fibrosis** | **2.6** |
| **43** | **89** | **Seroma** | **0.7** |
| **44** | **29** | **Fibroadenoma** | **0.8** |
| **45** | **33** | **Fibroadenoma** | **1.5** |
| **46** | **59** | **Apocrine Metaplasia + PASH + Stromal Fibrosis** | **1.0** |
| **47** | **47** | **Fibroadenoma** | **1.4** |
| **48** | **44** | **PASH** | **3.0** |
| **49** | **50** | **Cyst** | **0.8** |
| **50** | **56** | **Benign Nodule** | **3.6** |
| **51** | **36** | **Fibroadenoma** | **1.2** |
| **52** | **46** | **Fibroadenoma** | **0.7** |
| **53** | **54** | **Fibroadenoma** | **0.8** |
| **54** | **25** | **Fibroadenoma** | **1.7** |
| **55** | **51** | **Fibroadenoma** | **1.0** |
| **56** | **56** | **Cyst/Fibroadenoma** | **0.6** |
| **57** | **26** | **Fibroadenoma** | **2.3** |
| **58** | **49** | **Benign Nodule** | **2.7** |
| **59** | **39** | **Fibroadenoma** | **1.7** |
| **60** | **32** | **Benign Lipoma** | **3.9** |
| **61** | **20** | **Fibroadenoma** | **3.8** |
| **62** | **75** | **Cyst** | **1.4** |
| **63** | **53** | **Cyst** | **2.0** |
| **64** | **26** | **Fibroadenoma** | **2.1** |
| **65** | **44** | **Fibroadenoma** | **2.3** |
| **66** | **37** | **Fibroadenoma** | **0.8** |
| **67** | **45** | **Fibroadenoma** | **1.6** |
| **68** | **48** | **Cyst** | **2.2** |
| **69** | **53** | **Cyst** | **1.7** |
| **70** | **52** | **Fibroadenoma** | **1.1** |
| **71** | **47** | **Cyst** | **1.2** |
| **72** | **48** | **Cyst** | **1.6** |
| **73** | **63** | **Cyst** | **0.5** |
| **74** | **23** | **Fibroadenoma** | **1.6** |
| **75** | **52** | **Benign Nodule** | **1.1** |
| **76** | **46** | **Fibroadenoma** | **1.6** |
| **77** | **43** | **Fibroadenoma** | **1.6** |
| **78** | **61** | **Fibroadenoma** | **0.9** |
| **79** | **43** | **Cyst** | **2.0** |
| **80** | **37** | **Fibroadenoma** | **2.7** |
| **81** | **51** | **PASH** | **2.4** |
| **82** | **44** | **Fibroadenoma** | **1.0** |
| **83** | **30** | **Fibroadenoma** | **2.9** |
| **84** | **53** | **Cluster of Cysts** | **1.3** |
| **85** | **74** | **Cyst** | **1.4** |
| **86** | **39** | **Radial Scar** | **0.5** |
| **87** | **40** | **Fibroadenomatoid Changes** | **2.5** |
| **88** | **62** | **Benign Nodule** | **1.3** |
| **89** | **45** | **Cyst** | **1.0** |
| **90** | **48** | **Benign Nodule** | **1.6** |
| **91** | **49** | **Benign Nodule** | **1.1** |
| **92** | **50** | **Benign Nodule** | **1.4** |
