# Supplementary material for: Comparison of methods for texture analysis of QUS parametric images in the characterization of breast lesions
Source: PLoS One. 2020 Dec 31;15(12):e0244965. doi: 10.1371/journal.pone.0244965 (PMC7775053; doi:10.1371/journal.pone.0244965)
Supplement: S2 Table — Lesion size refers to the longest dimension of the tumor. ER is estrogen receptor, PR is progesterone receptor, HER2 is human epithelial growth factor receptor 2. Lesion size refers to the longest dimension of the tumor. IDC stands for invasive ductal carcinoma. ILC stands for invasive lobular carcinoma. DCIS stands for ductal carcinoma in situ, IMC stands for invasive mammary carcinoma. (DOCX) [file pone.0244965.s002.docx]

**S2 Table: Malignant Patient Characteristics.** Lesion size refers to the longest dimension of the tumor. ER is estrogen receptor, PR is progesterone receptor, HER2 is human epithelial growth factor receptor 2. Lesion size refers to the longest dimension of the tumor. IDC stands for invasive ductal carcinoma. ILC stands for invasive lobular carcinoma. DCIS stands for ductal carcinoma *in situ*, IMC stands for invasive mammary carcinoma.

| **Patient Number** | **Age** | **Type** | **Size (cm)** | **Tumor Grade** | **ER** | **PR** | **HER2** |
| --- | --- | --- | --- | --- | --- | --- | --- |
| **1** | **72** | **IDC** | **3.3** | **II** | **+** | **-** | **-** |
| **2** | **83** | **ILC** | **1.0** | **II** | **+** | **+** | **-** |
| **3** | **41** | **IDC** | **4.3** | **II/III** | **-** | **-** | **-** |
| **4** | **51** | **IDC** | **2.1** | **II** | **+** | **+** | **+** |
| **5** | **58** | **IDC** | **3.8** | **II/III** | **+** | **-** | **+** |
| **6** | **83** | **IDC** | **7.7** | **II/III** | **+** | **-** | **+** |
| **7** | **51** | **IDC** | **3.8** | **III** | **+** | **+** | **-** |
| **8** | **43** | **IDC** | **7.0** | **III** | **-** | **-** | **-** |
| **9** | **61** | **IDC** | **2.2** | **III** | **-** | **-** | **-** |
| **10** | **42** | **IDC** | **5.7** | **II/III** | **+** | **+** | **-** |
| **11** | **60** | **IDC** | **1.5** | **III** | **+** | **+** | **+** |
| **12** | **42** | **IDC** | **2.4** | **II/III** | **+** | **+** | **-** |
| **13** | **47** | **IDC** | **0.7** | **I** | **+** | **+** | **-** |
| **14** | **42** | **IDC** | **2.1** | **III** | **+** | **+** | **-** |
| **15** | **66** | **IDC** | **3.8** | **II** | **+** | **+** | **-** |
| **16** | **51** | **IDC** | **9.6** | **II/III** | **+** | **+** | **+** |
| **17** | **53** | **Invasive Carcinoma** | **3.5** | **II/III** | **-** | **-** | **-** |
| **18** | **69** | **IDC** | **0.7** | **II** | **+** | **+** | **-** |
| **19** | **45** | **IDC** | **3.5** | **II/III** | **+** | **+** | **+** |
| **20** | **48** | **IDC** | **2.2** | **III** | **-** | **-** | **-** |
| **21** | **61** | **IDC** | **2.0** | **III** | **+** | **+** | **+** |
| **22** | **77** | **ILC** | **1.5** | **N/A** | **+** | **-** | **-** |
| **23** | **63** | **DCIS** | **1.3** | **III** | **+** | **+** | **-** |
| **24** | **41** | **IDC** | **1.5** | **III** | **+** | **+** | **-** |
| **25** | **64** | **IDC** | **1.2** | **II** | **+** | **+** | **-** |
| **26** | **44** | **IDC/ILC** | **0.9** | **II** | **+** | **+** | **-** |
| **27** | **50** | **IDC** | **4.2** | **II** | **+** | **+** | **+** |
| **28** | **61** | **Invasive Adenocarcinoma** | **2.1** | **II** | **-** | **-** | **-** |
| **29** | **73** | **IDC** | **2.0** | **II** | **+** | **+** | **-** |
| **30** | **71** | **ILC** | **4.0** | **II** | **+** | **+** | **+** |
| **31** | **70** | **IDC** | **8.0** | **I** | **+** | **+** | **-** |
| **32** | **79** | **IDC** | **1.8** | **II** | **+** | **+** | **+** |
| **33** | **51** | **Invasive Tubular Carcinoma** | **1.8** | **I** | **+** | **+** | **-** |
| **34** | **43** | **IDC** | **1.5** | **II** | **+** | **+** | **+** |
| **35** | **70** | **IDC** | **1.7** | **I/II** | **+** | **+** | **-** |
| **36** | **56** | **IDC** | **5.0** | **II** | **+** | **+** | **-** |
| **37** | **47** | **IDC** | **2.6** | **III** | **+** | **+** | **+** |
| **38** | **63** | **IDC** | **8.6** | **II** | **+** | **+** | **-** |
| **39** | **52** | **IDC** | **3.1** | **II/III** | **-** | **-** | **-** |
| **40** | **54** | **IDC** | **2.3** | **III** | **-** | **-** | **-** |
| **41** | **68** | **IDC** | **2.2** | **II** | **+** | **+** | **+** |
| **42** | **49** | **IDC** | **2.5** | **II/III** | **-** | **-** | **-** |
| **43** | **49** | **IDC** | **2.6** | **II** | **+** | **+** | **+** |
| **44** | **60** | **IDC** | **6.0** | **III** | **-** | **-** | **+** |
| **45** | **57** | **IDC** | **3.3** | **III** | **-** | **-** | **+** |
| **46** | **57** | **IDC** | **2.1** | **N/A** | **+** | **-** | **+** |
| **47** | **67** | **IDC** | **2.5** | **III** | **+** | **+** | **-** |
| **48** | **55** | **IDC** | **3.2** | **III** | **-** | **-** | **+** |
| **49** | **45** | **IDC** | **2.6** | **II** | **+** | **+** | **-** |
| **50** | **45** | **Invasive Metaplastic Carcinoma** | **3.3** | **N/A** | **-** | **-** | **-** |
| **51** | **55** | **IDC** | **2.9** | **II** | **+** | **+** | **-** |
| **52** | **64** | **Malignant Neoplasm** | **3.3** | **N/A** | **-** | **-** | **-** |
| **53** | **42** | **IDC** | **2.1** | **III** | **+** | **+** | **+** |
| **54** | **38** | **IDC** | **1.9** | **III** | **-** | **-** | **+** |
| **55** | **72** | **IMC** | **5.8** | **III** | **-** | **-** | **-** |
| **56** | **36** | **IDC** | **2.3** | **II** | **+** | **+** | **+** |
| **57** | **27** | **IDC** | **1.5** | **II** | **+** | **+** | **+** |
| **58** | **36** | **IDC** | **N/A** | **II** | **+** | **+** | **-** |
| **59** | **35** | **DCIS** | **1.6** | **N/A** | **-** | **-** | **+** |
| **60** | **69** | **ILC** | **6.2** | **II** | **+** | **+** | **-** |
| **61** | **81** | **IMC** | **2.9** | **I** | **+** | **+** | **-** |
| **62** | **38** | **IMC** | **1.5** | **II** | **+** | **+** | **-** |
| **63** | **50** | **IDC** | **5.7** | **III** | **+** | **+** | **+** |
| **64** | **67** | **IDC** | **N/A** | **I, II, III** | **+** | **+** | **-** |
| **65** | **63** | **IDC** | **0.6** | **II** | **+** | **+** | **-** |
| **66** | **53** | **IDC** | **5.2** | **III** | **+** | **+** | **-** |
| **67** | **64** | **IDC** | **3.7** | **III** | **+** | **-** | **-** |
| **68** | **54** | **IDC** | **5.0** | **II** | **+** | **+** | **-** |
| **69** | **81** | **IDC** | **3.8** | **III** | **-** | **-** | **-** |
| **70** | **55** | **IMC** | **3.4** | **III** | **-** | **-** | **-** |
| **71** | **31** | **IDC** | **4.6** | **II** | **+** | **+** | **-** |
| **72** | **34** | **IDC** | **4.0** | **III** | **+** | **+** | **-** |
| **73** | **46** | **IMC** | **8.0** | **N/A** | **+** | **-** | **+** |
| **74** | **53** | **IDC** | **4.8** | **II** | **+** | **+** | **-** |
| **75** | **67** | **IDC** | **N/A** | **II** | **+** | **+** | **-** |
| **76** | **51** | **IMC** | **1.9** | **II** | **+** | **+** | **-** |
| **77** | **67** | **IDC** | **1.6** | **III** | **-** | **-** | **-** |
| **78** | **77** | **ILC** | **5.0** | **III** | **-** | **-** | **-** |
| **79** | **47** | **Locally-advanced Carcinoma** | **N/A** | **III** | **+** | **-** | **-** |
| **80** | **34** | **IDC** | **1.9** | **III** | **-** | **-** | **-** |
| **81** | **51** | **IDC** | **1.9** | **III** | **-** | **-** | **-** |
| **82** | **47** | **IDC** | **N/A** | **III** | **-** | **-** | **+** |
| **83** | **37** | **IDC** | **3.0** | **II** | **+** | **+** | **-** |
| **84** | **49** | **IDC** | **5.8** | **III** | **-** | **-** | **-** |
| **85** | **42** | **IDC** | **3.1** | **N/A** | **-** | **-** | **+** |
| **86** | **42** | **IDC** | **8.5** | **III** | **+** | **-** | **-** |
| **87** | **66** | **IDC** | **1.7** | **N/A** | **-** | **-** | **+** |
| **88** | **50** | **IDC** | **3.7** | **I-II** | **+** | **+** | **-** |
| **89** | **68** | **IMC** | **2.5** | **II** | **+** | **-** | **-** |
| **90** | **35** | **IDC** | **4.4** | **II** | **+** | **+** | **+** |
| **91** | **67** | **IDC** | **2.3** | **II** | **+** | **-** | **-** |
| **92** | **43** | **IDC** | **5.3** | **III** | **-** | **-** | **+** |
| **93** | **60** | **IDC** | **2.4** | **II** | **+** | **-** | **-** |
| **94** | **49** | **IDC** | **1.8** | **III** | **+** | **+** | **-** |
| **95** | **50** | **IDC** | **4.1** | **II** | **+** | **+** | **+** |
| **96** | **51** | **IDC** | **3.3** | **III** | **+** | **+** | **-** |
| **97** | **49** | **IDC** | **2.2** | **II** | **+** | **+** | **-** |
| **98** | **72** | **IDC** | **1.8** | **III** | **+** | **+** | **+** |
| **99** | **41** | **IDC** | **3.2** | **III** | **+** | **+** | **+** |
| **100** | **51** | **IDC** | **2.7** | **III** | **-** | **-** | **+** |
| **101** | **63** | **IDC** | **3.7** | **III** | **-** | **-** | **-** |
