# Supplementary material for: Comparison of methods for texture analysis of QUS parametric images in the characterization of breast lesions
Source: PLoS One. 2020 Dec 31;15(12):e0244965. doi: 10.1371/journal.pone.0244965 (PMC7775053; doi:10.1371/journal.pone.0244965)
Supplement: S3 Table — (DOCX) [file pone.0244965.s003.docx]

**S3 Table: BI-RADS Distribution among the lesions evaluated.**

| **BI-RADS Score** | **Number of Lesions** | **Benign** | **Malignant** |
| --- | --- | --- | --- |
| **1** | **3** | **3** | **0** |
| **2** | **22** | **22** | **0** |
| **3** | **31** | **31** | **0** |
| **4** | **41** | **35** | **6** |
| **5** | **51** | **0** | **51** |
| **6** | **43** | **0** | **43** |
| **N/A** | **2** | **1** | **1** |
| **Total** | **193** | **92** | **101** |
